# Exercise empowerment: a scoping review of randomized controlled trials and quasi-experimental physical activity interventions

**DOI:** 10.1186/s12966-025-01859-9

**Published:** 2025-12-10

**Authors:** Aspen E. Streetman, Rachel L. Walden, Colin J. Orr, William J. Heerman

**Affiliations:** 1 Vanderbilt University Medical Center, 2146 Belcourt Avenue, Nashville, TN 37212 USA; 2 Vanderbilt University Eskind Biomedical Library, 2209 Garland Avenue, Nashville, TN 37240 USA

**Keywords:** Health behavior change, PRISMA-ScR, Physical activity interventions, Randomized controlled trials, Theory, Autonomy, Agency

## Abstract

**Background:**

Physical inactivity is a leading contributor to chronic disease and premature mortality across the globe. Traditional physical activity interventions often promote physical activity initiation but have limited long-term effectiveness. Empowerment has been recognized as a potentially powerful tool in health behavior change, but it is unclear how empowerment is operationalized in physical activity interventions. One current gap in the literature is the role of empowerment in physical activity interventions. Therefore, this review aims to determine how exercise empowerment is defined, measured, and theoretically framed in randomized controlled trials and quasi-experiments across various populations. We are also interested in understanding the strategies for facilitating empowerment, whether empowering interventions increase physical activity, and if physical activity outcomes are assessed.

**Methods:**

A scoping review adhering to the Preferred Reporting Items for Systematic reviews and Meta-Analyses extension for Scoping Reviews was conducted across five electronic databases (PubMed, Embase, PsycINFO, Web of Science, Cochrane Library, CINAHL). Inclusion criteria included adults and children, participation in a physical activity intervention, and an empowerment strategy. Exclusion criteria included qualitative studies, studies published before 1994, and studies not in English.

**Results:**

Forty papers were included in this scoping review, but no interventions explicitly defined exercise empowerment. Empowerment was not directly measured in any of the included interventions. Empowerment theory or a derivative informed nine (22.5%) of the included articles, while six (15%) lacked theoretical underpinnings. Most interventions (55%) utilized an educational approach. Physical activity was measured subjectively in 27 (67.5%) interventions. Physical activity increased in most studies (*n* = 29; 72.5%).

**Conclusions:**

This review highlights the disconnect between the multidimensional framework of empowerment theory and interventions designed to empower participants. We identified several opportunities to increase intervention effectiveness, including developing and applying a consensus definition of exercise empowerment that creates better alignment with empowerment theory. Exercise empowerment should be defined as the perceived confidence and control over exercise choices that lead to sustained exercise and a sense of fulfillment. It can be experienced both individually and collectively. The process of building exercise empowerment depends on developing personal confidence and control over exercise choices, engaging with supportive social networks to initiate and sustain exercise, and utilizing available community-based exercise resources.

**Supplementary Information:**

The online version contains supplementary material available at 10.1186/s12966-025-01859-9.

## Background

Regular physical activity helps prevent and manage non-communicable diseases like heart disease, hypertension, stroke, diabetes, and several types of cancers [1–3]. It also provides life-enhancing benefits by helping individuals maintain a healthy weight and improve mental health [4–6]. Physical activity refers to all movement and includes exercise. Exercise is planned, structured, repetitive, and done to improve or maintain physical fitness [7]. Globally, current physical activity guidelines for adults include 150 min of mild to moderate physical activity and two full-body-focused muscle-strengthening sessions weekly [8, 9]. Guidelines for children and adolescents recommend 60 min of moderate to vigorous physical activity daily [8, 9]. However, current global estimates suggest that one out of four adults and 81% of adolescents do not do enough physical activity [9]. This lack of physical activity has significantly burdened the world’s economy, resulting in an estimated international dollar (INT$)53.8 billion in direct healthcare expenses and (INT$)13.7 billion in losses due to reduced productivity [10]. Evidence indicates a gap between knowledge and behavior regarding physical activity, as many individuals recognize its benefits, yet only a small percentage meet the physical activity guidelines [11–13].

Barriers to physical activity and exercise are complex, cutting across all levels of the social-ecological model [14–23]. Examples of individual-level barriers include low self-efficacy, lack of motivation, and fear of injury [19, 21]. Lack of support, family care responsibilities, and adherence to traditional cultural and gender norms are common interpersonal-level barriers to physical activity and exercise [14, 15]. Community-level barriers include access to safe spaces for physical activity and exercise, the high cost of gym memberships, fewer opportunities for organized physical activity, and limited access to recreational facilities [16, 17]. While physical activity and exercise barriers are well-described [18, 24–26], traditional health promotion approaches to increase physical activity have shown modest effectiveness, especially over time [22, 27]. One potential reason for their limited effectiveness is that they do not address the multiple barriers that influence sustained participation in physical activity.

Empowerment theory [28] conceptualizes empowerment as a multidimensional construct that may play a significant role in initiating and maintaining physical activity and exercise. According to empowerment theory, empowerment is both a process and an outcome in which individuals gain skills and capabilities to gain power and control [28, 29]. Empowerment theory applies empowerment to the individual, their friends and family, and the community in which they live. At the individual level, empowerment includes perceived control, critical awareness, and participatory behaviors. Individual-level empowerment is conceptually related to self-efficacy, autonomy, and agency, but it is a distinct behavior change construct. Self-efficacy refers to an individual’s belief in their ability to successfully perform a specific task [30]. Autonomy reflects a sense of choice, while agency encompasses confidence and control [31, 32]. Interpersonal empowerment focuses on shared leadership, skill development, and effective resource management. Community empowerment involves collective action. Empowerment has been operationalized in various populations, including among individuals with chronic disease and cancer, and has been shown to improve health outcomes through better medication adherence and lifestyle modification [33–40]. Exercise empowerment is an understudied construct that could be better utilized in health promotion interventions to increase physical activity. While some interventions describe themselves as empowering participants to do more physical activity, the term empowerment is often used descriptively rather than analytically, and it is often unclear how empowerment is operationalized or measured, leading to definitional and theoretical dilution [41, 42]. Additionally, empowerment-based interventions may not holistically target al.l three dimensions of empowerment theory, depending on the study’s purpose. For example, systematic reviews and meta-analyses have shown that individual-level empowerment concepts (e.g., self-efficacy, autonomy, motivation) have been widely used to increase physical activity effectively [43, 44], but these interventions fall short of fully utilizing empowerment’s holistic potential by primarily focusing on the individual level of empowerment. Interventions that focus on the interpersonal levels of empowerment have shown that addressing social empowerment dynamics, such as breaking down social barriers, challenging cultural or societal norms, and creating supportive community networks, can mitigate barriers to physical activity [45, 46]. When it comes to community-level empowerment factors, evidence suggests that addressing community-level empowerment may lead to increased access to resources, removal of economic and logistical challenges, development of supportive infrastructure, and the creation of inclusive environments for physical activity [47–51]. Despite the evidence linking empowerment to the improvement of chronic health conditions and evidence suggesting that increased empowerment may lead to more physical activity, the current literature lacks a comprehensive mapping of how exercise empowerment is defined, measured, and theoretically framed. Therefore, this scoping review aims to answer the following questions: (1) How is exercise empowerment defined, measured, and theoretically framed in randomized controlled trials (RCTs) and quasi-experimental studies? (2) What strategies facilitate empowerment in RCTs and quasi-experimental studies? (3) Do empowering interventions increase physical activity in RCTs and quasi-experiments? (4) Were physical activity outcomes assessed? This review is focused explicitly on RCTs and quasi-experiments, as RCTs provide the strongest evidence for causal relationships. However, including quasi-experiments allows for a broader examination of evidence while maintaining methodological rigor.

## Methods

### Protocol and registration

The primary object of this project was to identify how exercise empowerment is defined, measured, and theoretically framed in RCTs and quasi-experimental studies. Therefore, we conducted a systematic scoping review as it is a preferred review method for clarifying concepts and definitions while maintaining rigor [52, 53]. We followed the Preferred Reporting Items for Systematic Reviews and Meta-Analysis Systematic scoping review extension (PRISMA-ScR) guidelines [54]; the complete PRISMA-ScR checklist is available in Appendix 1. Furthermore, this review was registered in the Open Science Framework (10.17605/OSF.IO/VGSM9) to pre-define objectives, methods, and reporting.

A comprehensive search was conducted using PubMed (NLM), Embase (Elsevier), PsycINFO (ProQuest), Web of Science (Clarivate), and CINAHL (EBSCOhost) in October 2024 and again in April 2025. The search strategies were crafted with the assistance of a medical librarian using the Population, Intervention, Comparison, and Outcome (PICO) framework [53], see Table 1. Exclusion criteria included the following: (1) studies that reported only qualitative data, (2) studies that did not use quasi-experimental or randomized controlled trial methodology, (3) studies from 1994 and older, as empowerment as conceptualized by empowerment theory and used in this review was first defined in 1995 [28], (4) studies that were not peer-reviewed, and (5) studies that were not in English. Full search strategies are in Appendix 2.

**Table 1:** Description of the Population, Intervention, Comparison, outcome (PICO) criteria for study inclusion

PICO Tool	Description
Population	Individuals participating in exercise or physical activity interventions
Intervention	Exercise empowerment strategies or interventions
Comparison	Usual care, no intervention, or alternative exercise interventions without explicit empowerment components
Outcome	Physical activity levels or related outcomes

Two rounds of screening occurred in Covidence [55]. Covidence is a web-based collaboration software platform that streamlines the production of systematic and other literature reviews. First, two reviewers performed title and abstract screening. The first author screened all articles to provide consistency, while a team of 15 trained researchers participated in the title and abstract screening phase. Articles were coded as potentially eligible or ineligible. Second, the full text of articles marked as potentially eligible was reviewed by two reviewers to ensure they met eligibility requirements. After screening, data extraction was completed in Microsoft Excel [56] using a standard extraction form (see Appendix 3 for complete extraction for all included studies). These articles were individually analyzed to identify how exercise empowerment was defined, the theoretical frameworks guiding the interventions, the types of empowerment strategies used, the duration and intensity of empowerment interventions, and physical activity intervention outcomes. Parallel extraction for consistency was performed on ten interventions to ensure consistency.

### Analysis

Cohen’s Kappa was calculated in Covidence [55]. Descriptive statistics were calculated in Microsoft Excel [56] and are presented as means ± standard deviation, frequencies, and percentages.

## Results

**Fig. 1:**
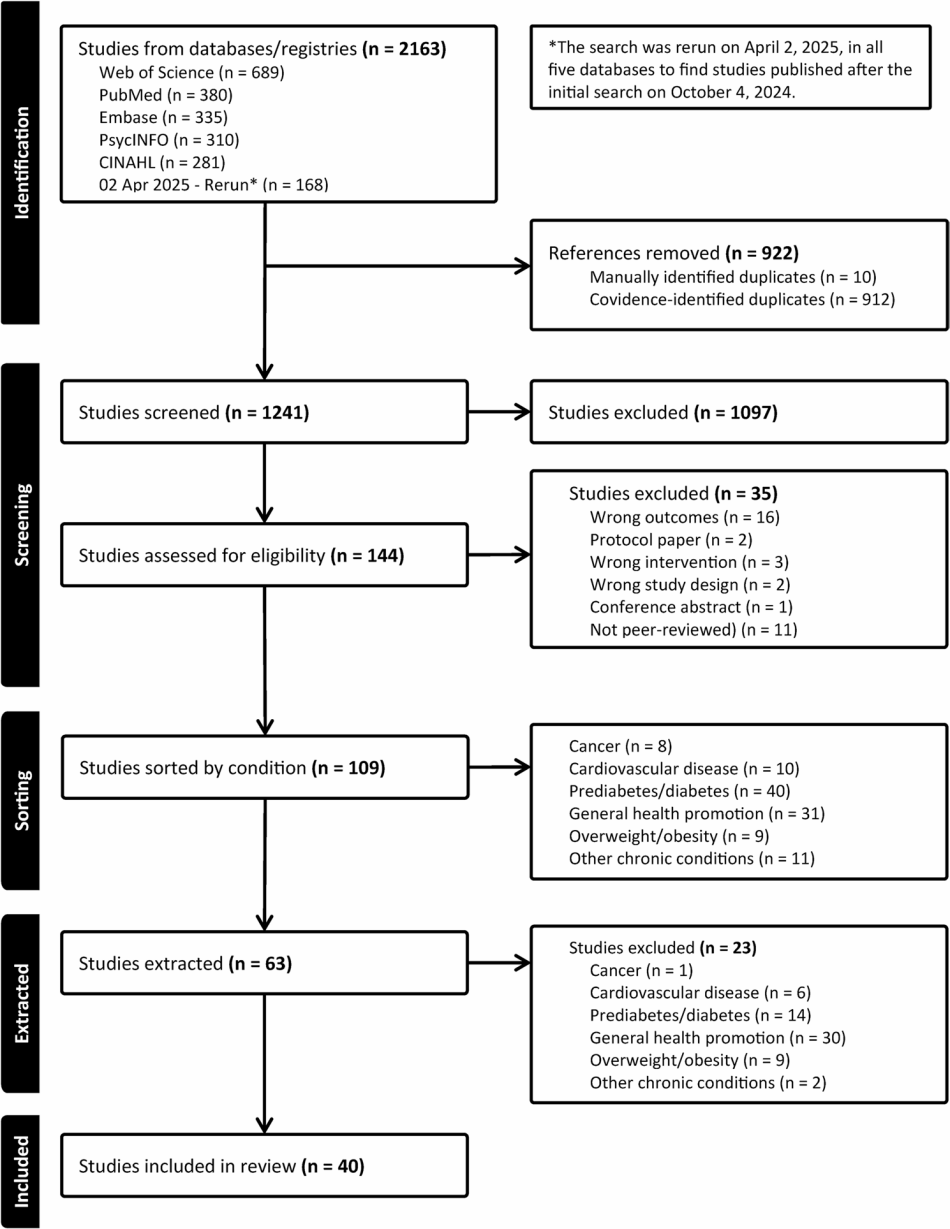
PRISMA-ScR flow diagram

A total of 2163 articles were identified. Of those, 922 were marked as duplicates (10 were identified manually, and Covidence identified 912), leaving 1241 articles. Title and abstract review eliminated 1020 articles. Interrater reliability expressed as Cohen’s Kappa averaged 0.55 ± 0.24, range 0.24–1.0.24.0, suggesting that, on average, agreement between reviewers was moderate. However, despite a moderate initial inter-rater reliability, consensus discussion resolved each discrepancy between reviewers. Reviewers met regularly to clarify inclusion criteria and adjudicate ambiguous cases. This process ensured that the final article inclusion reflected a shared understanding and agreement, despite variability in the initial ratings. One hundred forty-four articles were marked as potentially eligible for inclusion. Upon completing a full-text review, 109 articles remained. These articles were then grouped by the intervention population’s health condition or disease (cancer, *n* = 8; cardiovascular disease, *n* = 10; prediabetes/diabetes, *n* = 40; general health promotion, *n* = 31; overweight/obesity, *n* = 9; other chronic conditions, *n* = 11). We extracted data across all conditions before eliminating articles that included participants with chronic conditions or diseases (cancer, cardiovascular disease, prediabetes/diabetes, other chronic conditions), as these interventions aimed to promote health behaviors that support disease management through patient empowerment. Patient empowerment differed from exercise empowerment in that it broadly focused on patients gaining greater control over their healthcare-related decisions [57]. These interventions often employed an approach that provided information on the risks and benefits of participating in health-promoting activities, followed by support to achieve health-promoting goals. While this broad focus had some overlap with physical activity engagement, it was too broad for the scope of this review. Forty articles remained for analysis. One intervention, Dads and Daughters Exercising and Empowered (DADEE), was deployed and assessed in three articles [58–60]. The COPE (Creating Opportunities for Personal Empowerment) Healthy Lifestyles TEEN (Thinking, Emotions, Exercise, Nutrition) Program was deployed and assessed in two different articles [61, 62]. See Fig. 1 for the PRISMA flow diagram from abstract screening to final inclusion.

### Population characteristics

The number of participants varied considerably among the articles included, ranging from *N* = 21 to *N* = 5882. Adults (i.e., those aged 25–64) were the most frequently targeted population (*n* = 15; 37.5%), followed by youth aged 14–25 (*n* = 8; 20%). All studies’ majority sex was female (*n* = 40; 100%), and 13 studies (32.5%) were for women only. Nineteen articles (47.5%) did not report participants’ racial/ethnic group; see Table 2 for an overview of participant demographics.

**Table 2:** Participant demographics of the included studies (*N* = 40)

Participant Demographics	*n*	% (of total *N* = 40)
Sex		
Females only	13	32.5%
Mixed sexes	27	67.5%
Age		
Children (aged 0–14)	5	12.5%
Youth (aged 15–24)	8	20.0%
Adults (aged 24–64)	15	37.5%
Older Adults (aged 65+)	6	15.0%
More than one (parents/children)	6	15.0%
Children (aged 0–14)	5	12.5%
Race/Ethnicity Reported		
African American	7	17.5%
Hispanic/Latino	4	10.0%
Non-Hispanic White	7	17.5%
Other*	3	7.5%
Not reported	19	47.5%

*Other represents race/ethnicity represented in only one intervention; they are Australian, Iranian, and Japanese

### Article characteristics

The included articles were published from 2008 to 2024. The year with the most studies published was 2013 (*n* = 5; 12.5%). Among these, the majority (*n* = 20; 50%) of the interventions took place in the United States, used quasi-experimental methods (*n* = 26; 65%), and were publicly funded (*n* = 18; 45%), as shown in Table 3. Notably, only three middle-income countries were included. They were India, Mexico, and Serbia.

**Table 3:** Characteristics of the included studies (*N* = 40)

Study Characteristics	*n*	% (of total *N* = 40)
Study location^†^		
United States of America	20	50.0%
Australia	5	12.5%
Iran	3	7.5%
Indonesia	2	5.0%
Spain	3	7.5%
Sweden	2	5.0%
Other^#^	7	18.5%
Type of Experiment		
Quasi-experimental	26	65.0%
RCT	14	35.0%
Funding Source		
Not funded	2	5.0%
Not reported	10	25.0%
Private	9	22.5%
Public	18	45.0%
Both	1	2.5%
Type of intervention*		
Educational	22	55.0%
Educational and physical activity	8	20.0%
Community-based participatory action	8	20.0%
Coaching (research staff, peer, community health worker)	7	17.5%
Coaching and physical activity	1	2.5%
Technology-enhanced or based	7	17.5%
Policy change	2	5.0%
Gaming	1	2.5%
Media campaign	1	2.5%
Theoretical foundation ^+^		
Empowerment theory or derivative	9	22.5%
Health self-empowerment theory	2	5.0%
Social cognitive theory	13	32.5%
Self-determination theory	7	17.5%
Transtheoretical model	3	7.5%
Health belief theory	2	5.0%
Family ecological model	2	5.0%
No theory listed	6	15.0%
Other	12	30.0%

^†^One intervention was a multicenter study conducted in Italy, Serbia, and Spain. ^#^Other locations are those in which only one study was conducted. These locations are Italy, India, Mexico, Serbia, South Korea, Taiwan, and the United Kingdom. *Some studies relied on more than one strategy or technique. ^+^Some studies employed more than one theoretical approach

### Intervention characteristics

Intervention characteristics varied among the included interventions; see Table 3. Educational sessions (*n* = 22; 55%), educational sessions combined with physical activity (*n* = 8; 20%), and community-based participatory action research (*n* = 8; 20%) were the most commonly implemented empowerment strategies in the interventions reviewed.

### Theoretical foundation

Theory guided the majority (*n* = 34; 85%) of the included interventions, and some relied on more than one theory; see Table 3. However, empowerment theory or a derivative of empowerment theory (i.e., premenopausal empowerment model, empowerment model, family-centered model of empowerment) guided nine (22.5%) interventions. Two (5%) different interventions relied on health self-empowerment theory [63, 64]. Health self-empowerment theory is multidimensional and “recognizes that racial/ethnic minorities often experience intractable social, ecological, and cultural determinants of health that are often barriers to engaging in health-smart behaviors” [64]. Thus, these interventions aimed to empower participants to identify barriers to health behavior change at the individual, interpersonal, and community levels, similar to empowerment theory, but for a specific population [63, 64]. The ecosystemic structural family therapy model, BSmarter model, wellness motivation theory, integrative healthcare model, social learning theory, family-centered empowerment model, and premenopausal empowerment model were used in one intervention each (2.5%).

### Empowerment and physical activity outcomes

Table 4 provides an overview of empowerment and physical activity outcomes. Exercise empowerment was not directly measured in any of the included interventions. Most interventions increased physical activity (*n* = 29; 72.5%). Self-report questionnaires were used to measure physical activity in most interventions (*n* = 27; 67.5%). Thirteen (32.5%) interventions measured physical activity post-intervention. Among those, four (10%) followed up 12 months post-intervention, and one (2.5%) followed up more than 12 months post-intervention. The remaining follow-up timepoints were 2 months (*n* = 1; 2.5%), 3 months (*n* = 2; 5%), 6 months (*n* = 3; 7.5%), and 9 months (*n* = 2; 5%).

**Table 4:** Empowerment and physical activity-related outcomes of the included studies (*N* = 40)

Outcomes	*n*	% (of total *N* = 40)
Empowerment related		
Exercise empowerment	0	0.0%
Increased health empowerment	2	5.0%
Decreased or no change in parent empowerment	1	2.5%
Physical activity related		
Increased physical activity	29	72.5%
Did not increase physical activity	8	20.0%
Unable to determine changes in physical activity	3	7.5%
Measured subjectively	27	67.5%
Measured objectively	13	32.5%
Pedometer	7	17.5%
Accelerometry	6	15.0%

### Narrative conceptual analysis

#### Empowerment related to exercise

Exercise empowerment was not explicitly defined in any of the 40 articles that were included in this review. However, four articles implicitly described exercise empowerment as a multidimensional construct [65–68]. In one intervention, empowerment was presented as a multidimensional construct measured by women’s perceptions of their abilities to identify barriers, solutions, and opportunities related to physical activity behavior [66]. Women were encouraged to work as a group to change their personal practices and gain confidence in their abilities to advocate for change in their neighborhoods [66]. In another intervention among older adults, the authors noted, “Empowerment theories create a mechanism for community participation and capacity building. In older adults, especially, empowerment strategies can lead to increased agency for engaging in physical activity and, both directly and indirectly, to improvements in quality of life and depressive symptoms” [67]. One culturally tailored weight loss intervention for African American women ensured the availability of programming opportunities, provided helping hand support (i.e., emotional reassurance, connecting individuals with resources), and offered training strategies to help individuals overcome barriers to physical activity [65]. This multidimensional approach aims to empower individuals to acquire new skills and utilize resources to gain control, while uniting marginalized communities to leverage their strengths and resources to improve their quality of life [65]. The Yoga for HEART conceptual model operationalized social support, environmental resources, self-knowledge, motivational appraisal, and self-regulation to result in participation in moderate-intensity physical activity [68].

#### Empowerment mentioned but not defined

Nine articles mentioned empowerment without clearly defining it [58–60, 69–74]. For example, the DADEE intervention aimed to empower fathers and daughters to combat societal gender bias that restricts girls’ participation in physical activity but did not define it [58–60]. In another article, empowerment was used once in the abstract, though the intervention aimed to foster autonomy by allowing school-level leaders to select intervention components in alignment with the school’s perceived needs to increase physical activity among students [72]. The Pasos Adelante intervention aimed to promote personal and community empowerment, but did not define empowerment [74].

### Synthesis of results

We identified forty articles that met our inclusion criteria and found that exercise empowerment was not explicitly defined in any of the included articles. Instead, the meaning of exercise empowerment was often implied. Exercise empowerment was not directly measured in the included articles. The theoretical underpinnings varied widely across these articles. Most interventions that used empowerment to describe their approach relied on educational strategies to facilitate it. Physical activity outcomes were measured subjectively, using self-report questionnaires in most interventions. The majority of interventions increased physical activity.

## Discussion

This scoping review identified several interventions that used empowerment as a core strategy to increase physical activity. However, none of the interventions explicitly defined exercise empowerment, which suggests several opportunities for future intervention development, evaluation, and policy change. Specifically, operationalizing a consensus definition of exercise empowerment would allow for better alignment with empowerment theory and facilitate the direct measurement of exercise empowerment. Establishing a clear and universally accepted definition of exercise empowerment would allow researchers, practitioners, and policymakers to systematically integrate empowerment principles into comprehensive intervention strategies, enhance empowerment mechanisms, evaluate program effectiveness, and develop policies that prioritize equitable access to empowering exercise opportunities, especially among traditionally underserved populations. Clearly defining exercise empowerment has the potential to shift the focus from short-term behavior change to building lasting capacity for self-management and intrinsic motivation through cognitive and behavioral mechanisms.

Our findings align with previous research suggesting that the meaning of empowerment becomes diluted when it is not explicitly defined or aligned with empowerment theory [41, 42]. We found some interventions used empowerment descriptively rather than analytically. While most interventions included in this review had theoretical underpinnings, the majority were not grounded in empowerment theory. Therefore, most interventions were not in alignment with all domains (individual, interpersonal, and community) of empowerment. Without a clear connection to empowerment theory, the term “empowerment” risks being used inconsistently and often reduces to general ideas of self-efficacy. This lack of precision can hinder the development of interventions that deliberately promote empowerment. It also makes evaluation more challenging and obscures the impact of empowerment on outcomes. Clearly defining exercise empowerment and anchoring it to empowerment theory can ensure conceptual clarity, improve measurement accuracy, and increase the relevance of findings for practice and policy.

With this in mind, we propose a consensus definition of exercise empowerment that is consistent with empowerment theory (see Table 5). Exercise empowerment is the perceived confidence and control over exercise choices that lead to sustained exercise and a sense of fulfillment. It can be experienced both individually and collectively. The process of building exercise empowerment depends on developing personal confidence and control over exercise choices, engaging with supportive social networks to initiate and sustain exercise, and utilizing available community-based exercise resources. While we acknowledge that this definition overlaps with several other health behavior change theories and includes self-efficacy, agency, and resilience constructs reported in the interventions included in this review, we believe establishing a shared understanding of what constitutes exercise empowerment is foundational for advancing both physical activity promotion research and practice. Establishing a consensus definition enables researchers, practitioners, and policymakers to align core components (i.e., confidence and control, engaging with supportive social networks, and utilizing community-based exercise resources) that are consistent with empowerment theory. Future research should examine how these constructs relate to the initiation and maintenance of physical activity.

**Table 5:** Exercise empowerment by comparing it with empowerment theory and cross-checking it with other behavior change theories

Empowerment Theory Domain	Empowerment Theory	Our Definition	Example	Overlap with Other Theories
Individual	Development of perceived control and critical awareness. Can lead to participatory behaviors.	Building perceived confidence and control over physical activity choices.	Going for a run because you are intrinsically motivated to be physically active through knowledge acquisition.	Self-determination theory, social cognitive theory, health belief model, self-efficacy theory, social learning theory, transtheoretical stages of change, relapse prevention, decision balance.
Interpersonal	Group leadership, skill development, and effective resource management.	Engaging with supportive social networks to initiate and maintain physical activity.	Participating in a running group by regularly attending group runs for an extended period.	Social cognitive theory, the theory of planned behavior, social network theory.
Community	Collective action and to access government and other community resources.	Participating in collective physical activity using available community resources.	Organizing a running group and inviting friends to join. Utilizing community resources to facilitate your running group.	Socioecological model, community organization model.

Most interventions included in this review adopted an educational intervention strategy aligned with social cognitive theory to encourage physical activity. Therefore, most of the interventions reviewed focused on the cognitive aspects of behavior change more than on the behavioral ones. Solely focusing on the cognitive aspects of behavior change is problematic, as evidenced by the small number of individuals who meet the physical activity guidelines globally [8, 9]. Previous research has long documented the knowledge behavior gap, indicating that increased awareness does not always translate to adherence to physical activity guidelines [11–13]. Moreover, previous research suggests that education-only approaches typically fail to address key barriers to physical activity initiation [75, 76]. We contend that empowerment-based interventions should focus on both the cognitive and behavioral components of behavior change. Specifically, exercise empowerment interventions should include educational and physical activity-based activities to be as potent as possible. For example, programs might pair structured exercise performed in a group with educational sessions that build knowledge related to the benefits of physical activity, goal setting, and self-regulation strategies, as well as access to community spaces for physical activity and exercise. Such interventions could also utilize an educational dashboard to reinforce key concepts and skills introduced in the sessions, allowing personalized learning opportunities, progress tracking, and ongoing access to resources. Not only would this approach better align with empowerment theory, but it would also increase an individual’s likelihood of adopting and maintaining physical activity.

This review identifies opportunities to improve how exercise empowerment is measured. To our knowledge, there are two existing scales of exercise empowerment: the Empowering and Disempowering Motivational Climates Questionnaire and the Empowerment in Exercise Scale [77, 78]. While both have demonstrated strong psychometric properties across diverse populations and settings, they are primarily situated within the context of sport and group exercise [77, 78]. While these measures are valuable, they may not fully capture the dimensions of empowerment relevant to health promotion interventions. This inability to measure exercise empowerment directly in health promotion contexts represents a significant gap in the literature. In some cases, self-efficacy measures were used in studies as proxies of exercise empowerment, recognizing the overlap of these constructs [29, 66, 79–81]. Adopting a consensus definition of exercise empowerment is a necessary precursor to developing and validating tools specifically designed to measure exercise empowerment, rather than relying on proxy measurements. Creating and validating a scale that captures the core concepts of empowerment theory, as outlined in this review, should involve a rigorous, multi-step process [82] and may include measures of confidence, control, social support, knowledge of, as well as the ability to access, community resources. Direct measurement of exercise empowerment could improve the evaluation of health promotion programs by assessing both psychosocial outcomes and physical activity. It could also inform guideline development by including strategies that explicitly promote it, such as participatory research or customized goal setting. Finally, exercise empowerment data could guide policies aimed at increasing equitable access to exercise opportunities.

This review also emphasizes the importance of objectively assessing physical activity over time. While most of the included interventions demonstrated increased physical activity, most used self-report questionnaires, which have been shown to over-report physical activity [83–85]. Relying on self-reported measurements has the potential to skew data and affect results. These findings emphasize the importance of using objective measures, such as accelerometry, to assess physical activity outcomes and, thus, intervention effectiveness. Most interventions did not collect follow-up physical activity data. Empowerment theory dictates that empowerment is not just an outcome, but also a process [28, 29]. Therefore, without follow-up physical activity data, the delayed effects of increased empowerment and, consequently, the intervention were not captured [17, 22, 23]. Therefore, we recommend assessing physical activity outcomes objectively at least six months after the intervention ends to evaluate the sustainability of behavior change beyond the intervention’s immediate effects. Evidence supports that physical activity gains decline after six months, making it a critical point for assessing maintenance [22, 86, 87]. are ideal for providing further insight into long-term adherence, the six-month mark offers a practical and evidence-based interval for detecting physical activity maintenance. This approach will provide a more robust understanding of exercise empowerment and intervention effectiveness, especially if objective measurement methods are utilized. Moreover, a strength of exercise empowerment, as conceptualized in this review, is how empowerment catalyzes the initiation of physical activity and helps to maintain it in the long term.

### Methodological considerations

Several limitations should be acknowledged. Despite employing a broad search strategy overseen by a medical librarian across multiple databases, some relevant articles may have been overlooked, particularly since the results were limited to articles written in English. To enhance the rigor of the included studies, we concentrated the review on RCTs and quasi-experiments. However, we did not directly assess the quality of the studies. Our focus on RCTs and quasi-experimental studies may have precluded emerging or nuanced conceptualizations of exercise empowerment, due to a lag between conceptual innovation and empirical application. We also note that the interventions included in this review predominantly feature female, United States-based participants, which may limit broader generalizability. Lastly, researcher subjectivity in inclusion decisions and data charting could have introduced bias. 

## Conclusions

These findings highlight the need for a consensus definition of exercise empowerment that aligns with the multidimensional nature of empowerment theory. Future research to empower individuals to do more physical activity should operationalize the definition suggested in this review. Furthermore, future research should employ objective measurements of physical activity during and after the intervention concludes to evaluate changes in physical activity associated with empowerment more accurately.

## Supplementary Information

Supplementary Material 1. [88–106].

## Data Availability

The datasets used and/or analyzed for the current study are available from the corresponding author on reasonable request.

## Abbreviations

INT$: International dollar
RCT: Randomized controlled trial
PRISMA-ScR: Preferred Reporting Items for Systematic Reviews and Meta-Analysis Systematic scoping review extension
NLM: National Library of Medicine
CINAHL: Cumulative Index to Nursing and Allied Health Literature
PICO: Population, Intervention, Comparison, Outcome
DADEE: Dads And Daughters Exercising and Empowered
COPE: Creating Opportunities for Personal Empowerment Healthy Lifestyles TEEN Thinking, Emotions, Exercise, Nutrition Program
NIH: National Institutes of Health
NIDDK: National Institute of Diabetes and Digestive and Kidney Diseases
