# Supplementary material for: Exercise empowerment: a scoping review of randomized controlled trials and quasi-experimental physical activity interventions
Source: Int J Behav Nutr Phys Act. 2025 Dec 10;23:5. doi: 10.1186/s12966-025-01859-9 (PMC12821911; doi:10.1186/s12966-025-01859-9)
Supplement: Supplementary file 1 — Supplementary Material 1. [88–106]. [file 12966_2025_1859_MOESM1_ESM.docx]

**Appendices**

**Appendix 1.** Preferred Reporting Items for Systematic reviews and Meta-Analyses extension for Scoping Reviews (PRISMA-ScR) Checklist.

| **SECTION** | **ITEM** | **PRISMA-ScR CHECKLIST ITEM** | **REPORTED ON PAGE #** |
| --- | --- | --- | --- |
| **TITLE** | | | |
| Title | 1 | Identify the report as a scoping review. | 1 |
| **ABSTRACT** | | | |
| Structured summary | 2 | Provide a structured summary that includes (as applicable): background, objectives, eligibility criteria, sources of evidence, charting methods, results, and conclusions that relate to the review questions and objectives. | 2 |
| **INTRODUCTION** | | | |
| Rationale | 3 | Describe the rationale for the review in the context of what is already known. Explain why the review questions/objectives lend themselves to a scoping review approach. | 6-7 |
| Objectives | 4 | Provide an explicit statement of the questions and objectives being addressed with reference to their key elements (e.g., population or participants, concepts, and context) or other relevant key elements used to conceptualize the review questions and/or objectives. | 6-7 |
| **METHODS** | | | |
| Protocol and registration | 5 | Indicate whether a review protocol exists; state if and where it can be accessed (e.g., a Web address); and if available, provide registration information, including the registration number. | 7 |
| Eligibility criteria | 6 | Specify characteristics of the sources of evidence used as eligibility criteria (e.g., years considered, language, and publication status), and provide a rationale. | 8 |
| Information sources* | 7 | Describe all information sources in the search (e.g., databases with dates of coverage and contact with authors to identify additional sources), as well as the date the most recent search was executed. | 8 |
| Search | 8 | Present the full electronic search strategy for at least 1 database, including any limits used, such that it could be repeated. | Appendix 2 |
| Selection of sources of evidence† | 9 | State the process for selecting sources of evidence (i.e., screening and eligibility) included in the scoping review. | 8-9 |
| Data charting process‡ | 10 | Describe the methods of charting data from the included sources of evidence (e.g., calibrated forms or forms that have been tested by the team before their use, and whether data charting was done independently or in duplicate) and any processes for obtaining and confirming data from investigators. | 8-9 |
| Data items | 11 | List and define all variables for which data were sought and any assumptions and simplifications made. | 8 |
| Critical appraisal of individual sources of evidence§ | 12 | If done, provide a rationale for conducting a critical appraisal of included sources of evidence; describe the methods used and how this information was used in any data synthesis (if appropriate). | n/a |
| Synthesis of results | 13 | Describe the methods of handling and summarizing the data that were charted. | 8-9 |
| **RESULTS** | | | |
| Selection of sources of evidence | 14 | Give numbers of sources of evidence screened, assessed for eligibility, and included in the review, with reasons for exclusions at each stage, ideally using a flow diagram. | 10 |
| Characteristics of sources of evidence | 15 | For each source of evidence, present characteristics for which data were charted and provide the citations. | 10-11 |
| Critical appraisal within sources of evidence | 16 | If done, present data on critical appraisal of included sources of evidence (see item 12). | n/a |
| Results of individual sources of evidence | 17 | For each included source of evidence, present the relevant data that were charted that relate to the review questions and objectives. | 9-14 |
| Synthesis of results | 18 | Summarize and/or present the charting results as they relate to the review questions and objectives. | 14-15 |
| **DISCUSSION** | | | |
| Summary of evidence | 19 | Summarize the main results (including an overview of concepts, themes, and types of evidence available), link to the review questions and objectives, and consider the relevance to key groups. | 15 |
| Limitations | 20 | Discuss the limitations of the scoping review process. | 21 |
| Conclusions | 21 | Provide a general interpretation of the results with respect to the review questions and objectives, as well as potential implications and/or next steps. | 21 |
| **FUNDING** | | | |
| Funding | 22 | Describe sources of funding for the included sources of evidence, as well as sources of funding for the scoping review. Describe the role of the funders of the scoping review. | 22-23 |

JBI = Joanna Briggs Institute; PRISMA-ScR = Preferred Reporting Items for Systematic reviews and Meta-Analyses extension for Scoping Reviews.

* Where *sources of evidence* (see second footnote) are compiled from, such as bibliographic databases, social media platforms, and Web sites.

† A more inclusive/heterogeneous term used to account for the different types of evidence or data sources (e.g., quantitative and/or qualitative research, expert opinion, and policy documents) that may be eligible in a scoping review as opposed to only studies. This is not to be confused with *information sources* (see first footnote).

‡ The frameworks by Arksey and O’Malley (6) and Levac and colleagues (7) and the JBI guidance (4, 5) refer to the process of data extraction in a scoping review as data charting*.*

§ The process of systematically examining research evidence to assess its validity, results, and relevance before using it to inform a decision. This term is used for items 12 and 19 instead of "risk of bias" (which is more applicable to systematic reviews of interventions) to include and acknowledge the various sources of evidence that may be used in a scoping review (e.g., quantitative and/or qualitative research, expert opinion, and policy document).

*From:* Tricco AC, Lillie E, Zarin W, O'Brien KK, Colquhoun H, Levac D, et al. PRISMA Extension for Scoping Reviews (PRISMAScR): Checklist and Explanation. Ann Intern Med. 2018;169:467–473. [doi: 10.7326/M18-0850](http://annals.org/aim/fullarticle/2700389/prisma-extension-scoping-reviews-prisma-scr-checklist-explanation).

**Appendix 2.** Full search strategies.

**PubMed (NLM)**
Date Searched: 10/4/2024
Results Found: 380

| **Search** | **Query** |
| --- | --- |
| 1 | "exercise"[MeSH] |
| 2 | exercise[tiab] OR physical activit*[tiab] |
| 3 | 1 OR 2 |
| 4 | "empowerment"[MeSH] |
| 5 | empower*[tiab] |
| 6 | 4 OR 5 |
| 7 | “randomized controlled trial”[pt] OR “controlled clinical trial”[pt] |
| 8 | "Controlled Clinical Trials as Topic"[Mesh] |
| 9 | randomized[tiab] OR placebo[tiab] OR randomly[tiab] OR trial[ti] OR Quasi Experimental[tiab] OR non-equivalent control group[tiab] OR Quasi Experiment*[tiab] OR Intervention Study[tiab] |
| 10 | 7 OR 8 OR 9 |
| 11 | 3 AND 6 AND 10 |
| 12 | 11 NOT (animals [mh] NOT humans [mh]) |
| 13 | 12 AND English Language |

**Embase (Elsevier)**Date Searched: 10/04/2024
Results Found: 335

| **Search** | **Query** |
| --- | --- |
| 1 | 'exercise'/exp |
| 2 | 'exercise':ti,ab OR 'physical activit*':ti,ab |
| 3 | 1 OR 2 |
| 4 | 'empowerment'/exp |
| 5 | empower*':ti,ab |
| 6 | 4 OR 5 |
| 7 | 'randomized controlled trial':it OR 'controlled clinical trial':it |
| 8 | 'controlled clinical trial (topic)'/exp |
| 9 | 'randomized':ti,ab OR 'placebo':ti,ab OR 'randomly':ti,ab OR 'trial':ti OR 'quasi experimental':ti,ab OR 'non-equivalent control group':ti,ab OR 'quasi experiment*':ti,ab OR 'intervention study':ti,ab |
| 10 | 7 OR 8 OR 9 |
| 11 | 3 AND 6 AND 10 |
| 12 | 11 NOT ([animals]/lim NOT [humans]/lim) |
| 13 | 12 AND ([article]/lim OR [article in press]/lim OR [preprint]/lim) AND [english]/lim |

**APA PsycINFO (ProQuest)**Date Searched: 10/04/2024
Results Found: 310

| **Search** | **Query** |
| --- | --- |
| 1 | MAINSUBJECT.EXACT.EXPLODE("Exercise") |
| 2 | tiab(exercise OR physical activity) |
| 3 | 1 OR 2 |
| 4 | MAINSUBJECT.EXACT.EXPLODE("Empowerment") |
| 5 | tiab(empower*) |
| 6 | 4 OR 5 |
| 7 | MAINSUBJECT.EXACT.EXPLODE("Randomized Controlled Trials") |
| 8 | tiab(randomized OR placebo OR randomly OR Quasi Experimental OR non-equivalent control group OR Quasi Experiment* OR Intervention Study) |
| 9 | ti(trial) |
| 10 | 7 OR 8 OR 9 |
| 11 | 3 AND 6 AND 10 |
| 12 | 11 AND English Language |

**Web of Science (Clarivate)**Date Searched: 10/04/2024
Results Found: 689

| **Search** | **Query** |
| --- | --- |
| 1 | TS=(exercise OR physical activit*) |
| 2 | TS=(empower*) |
| 3 | TS=(randomized OR placebo OR Controlled OR randomly OR trial OR “Quasi Experimental” OR “non-equivalent control group” OR “Quasi Experiment*” OR “Intervention Study”) |
| 4 | 1 AND 2 AND 3 |
| 5 | 5 AND LA=(English) AND DT=(Article OR Early Access) |

**CINAHL (EBSCOhost)**Date Searched: 10/04/2024
Results Found: 281

| **Search** | **Query** |
| --- | --- |
| 1 | (MH "Exercise+") |
| 2 | “exercise” OR “physical activit*” |
| 3 | 1 OR 2 |
| 4 | (MH "Empowerment") |
| 5 | “Empower*” |
| 6 | 4 OR 5 |
| 7 | (MH "Intervention Trials") OR (MH "Nonrandomized Trials") |
| 8 | “randomized” OR “placebo” OR “Controlled” OR “randomly” OR “trial” OR “Quasi Experimental” OR “non-equivalent control group” OR “Quasi Experiment*” OR “Intervention Study”) |
| 9 | 7 OR 8 |
| 10 | 3 AND 6 AND 9 |
| 11 | 10 AND English Language |

**Appendix 3.** Complete extraction for all included studies.

| Author | Intervention name | Study type | Funding source | Study location | Intervention location | *N* | Age (years), range or M ± (SD) | Percent female | Majority racial/  ethnic group | Percent majority racial/  ethnic group | Exercise empowerment definition | Theoretical underpinning | Intervention approach | Measurement timepoints | Physical activity measure-ment | Physical activity outcome | Exercise empowerment measurement or proxy | Exercise empower-ment measurement or proxy outcome |
| --- | --- | --- | --- | --- | --- | --- | --- | --- | --- | --- | --- | --- | --- | --- | --- | --- | --- | --- |
| Ashton 2024 (59) | DADEE | QE | Publicly funded | Australia | After hours at a primary school | 542 | Daughters 7.7 (1.9) Fathers 40 (9.2) | Not reported | Non-Hispanic White | 98.8 | Definition not stated. The program aimed to combat societal gender bias that restricts girls' participation in sports and physical activity. Monitoring and control were integrated into the intervention. | Self-determination theory, social cognitive theory | Educational with physical activity | baseline, end of intervention, 12-month follow-up | Subjective | Increased | Not measured | n/a |
| Backman 2011 (67) | n/a | QE | Not reported | USA | Community-based organizations and direct health service provider sites. | 327 | 37-64 | 100 | African American | 100 | Not stated explicitly as physical activity or exercise empowerment. Empowerment was measured by women's perceptions of their ability to identify barriers, solutions, and opportunities regarding physical activity behavior. Moreover, empowerment was presented as a multidimensional construct in that women were encouraged as a group to change their personal practices and gain confidence in their abilities to advocate for change in their neighborhoods. | Social cognitive theory, the community-organizing principle of empowerment | Educational | baseline, end of intervention | Subjective | Increased | Health empowerment questions created for the intervention, describing physical activity barriers and facilitators | Increased |
| Baird 2014 (80) | Healthy Conversations Skills | QE | Publicly funded | UK | Pre-school | 512 | 31.81 (5.94) | 100 | Not reported | Not reported | Definition not stated. A patient empowerment approach was used by providing information on the risks and benefits of health behaviors, combined with goal setting and continued support. | None mentioned | Coaching | baseline, 12-month follow-up | Subjective | Increased | Efficacy for exercising | Did not increase |
| Barroso 2021 (89) | n/a | RCT | Publicly funded | Spain | Not reported (maybe primary care clinic) | 953 | 50 (10) | Not reported | Not reported | Not reported | Definition not stated. The intervention used an individually tailored approach to promote healthy behavior by encouraging participants to take responsibility for their own health and well-being. | Health belief model | Educational | baseline, 12-month follow-up | Subjective | Did not increase | Not measured | n/a |
| Barrows 2022 (69) | Yoga for Health Empowerment and Realizing Transformation (HEART) | RCT | Publicly funded | USA | Community yoga studio | 15 | 65 (8.5) | 93 | Non-Hispanic White | 100 | The Yoga for HEART conceptual model includes social support, environmental resources, self-knowledge, motivational appraisal, and self-regulation. Mobilizing these mechanisms of action results in participation in moderate-intensity physical activity. | Wellness motivation theory | Educational with physical activity | baseline, end of intervention | Objective | Did not increase | Possible selves questionnaire, self-efficacy subscale | Increased |
| Boudreau 2013 (90) | Power Up | RCT | Privately funded | USA | Community health center | 26 | Children: 9-12 Parents: not reported | Not reported | Hispanic/Latino(a) | 100 | Definition not stated. The ESFT Model is designed to enhance patient communication and compliance. | Ecosystem structural family therapy model | Educational, coaching | baseline, 6-month follow up | Objective | Did not increase | Not measured | n/a |
| Carels 2008 (74) | n/a | RCT | Not provided | USA | Not reported | 38 | 50.2 (9.9) | 84.2 | Non-Hispanic White | 88.6 | Definition not stated. Empowerment is used two times in the manuscript, but it is not defined. Similarly, a theoretical framework is not used. | None mentioned | Educational, coaching | baseline, end of intervention | Subjective | Increased | Not measured | n/a |
| Carvajal 2018 (75) | Pasos Adelante (Meta Salud was also reported, but no physical activity outcomes were available) | QE | Publicly funded | USA | Not reported | 247 | 51.6 (14.1) | 90 | Hispanic/Latino(a) | 88 | Definition not stated. The intervention aimed to promote personal and community empowerment. The LEARN Program for weight management is referenced. The LEARN Program is "A comprehensive self-help manual that gives you step-by-step instructions on how to lose weight and successfully maintain your weight loss." | Social cognitive theory | Educational, coaching | baseline, end of intervention, 3-month follow-up | Subjective | Did not increase | Not measured | n/a |
| Chandler 2015 (91) | Empower Resilience Intervention | QE | Publicly funded | USA | Web-based | 28 | 18-20 | 100 | Non-Hispanic White | 78 | Definition not stated. This intervention focused on fostering resilience through active coping, strength-building, cognitive flexibility, and social support. | Resilience training and positive youth development | Educational | baseline, end of intervention | Subjective | Increased | Not measured | n/a |
| Crist 2022 (68) | Peer Empowerment Program 4 Physical Activity (PEP4physical activity) | RCT | Publicly funded | USA | Senior/community centers | 476 | 71.0 (8.9) | 75.7 | Non-Hispanic White | 62.4 | "Empowerment theories create a mechanism for community participation and capacity building. In older adults, especially, empowerment strategies can lead to increased agency for engaging in PA and, both directly and indirectly, to improvements in quality-of-life and depressive symptoms." | Empowerment theory (Zimmerman, 1995), social cognitive theory, social ecological model | Coaching with physical activity | baseline, end of intervention | Objective | Increased | Not measured | n/a |
| Davison 2013 (92) | Communities for Healthy Living | QE | Publicly funded | USA | Pre-school | 154 | Children: 2-5 Parents: not reported | Not reported | Non-Hispanic White | Not reported | Definition not stated. Empowerment "results from understanding the forces that affect life situations (i.e., critical consciousness) and the ability to control these forces using resources and social support gained through social capital networks. Empowerment is fostered through critical reflection and equitable collaboration, resulting in increased power and resource redistribution." | Family ecological model and empowerment theory (Israel et al., 1994) | Educational, community-based participatory research, coaching | baseline, end of intervention | Objective | Increased | Not measured | n/a |
| Dewi 2023 (93) | From Fat to Fit with SMART Program | RCT | Publicly funded | Indonesia | College campus | 41 | 18-24 | Not reported | Not reported | Not reported | "Self-empowerment for students is an approach that involves the competence of individuals, in this case, students, in self-confidence, skill development, and participatory behavior." | Based on the BSMARTR coaching model | Educational, coaching | baseline, end of intervention | Subjective | Increased | Not measured | n/a |
| Doubova 2013 (94) | n/a | QE | Publicly funded | Mexico | Family medicine clinics | 380 | 49.3 (3.1) | 100 | Not reported | Not reported | Definition not stated. Empowerment was based on patient empowerment, an approach that aims to develop the patient's ability to identify problems (health needs), set goals related to health self-care, identify personal and social obstacles, develop solutions, and make appropriate decisions for self-care. | Integrative healthcare model with an empowerment approach | Educational | baseline, 6-month follow up | Subjective | Increased | Not measured | n/a |
| Ferrando-Terradez 2025 (95) | Women Involvement in Steady Exercise (WISE) | QE | Publicly funded | Spain, Italy, Serbia | Web-based | 61 | 20.1 (2.7) | 100 | Not reported | Not reported | Definition not stated. Empowerment was conceptualized as psychological (individual) empowerment. “Psychological empowerment is a multifaceted construct that includes intrapersonal components (perceptions of control, self-efficacy, and social support), interactional components (critical awareness of one’s environment), and behavioral components (actions taken to influence outcomes and exert control in a given context).” | Empowerment theory (Perkins & Zimmerman, 1995), self-determination theory | Educational, technology | baseline, end of intervention | Subjective | Increased | Not measured | n/a |
| Fröberg 2018 (49) | How-to-Act? Project | QE | Publicly funded | Sweden | Middle school | 114 | 12.8 (0.5) | 57.9 | Not reported | Not reported | Definition not stated. Empowerment was conceptualized as providing possibilities to formulate and influence opportunities and barriers for change and procuring motivation and belief in one’s ability. The intervention embraced the ideas of empowerment as both a goal and a process. | Empowerment (Tengland, 2007) | Educational, community-based participatory research | baseline, end of intervention | Objective | Did not increase | Not measured | n/a |
| Gago 2023 (81) | Communities for Healthy Living | RCT | Publicly and privately funded | USA | Pre-school | 3750 | Children: 2-5 Parents: not reported | 49.1 | Not reported | 41.2 (parents) | Definition not stated. The intervention's theory of change integrates the family ecological model and empowerment theory. | Family ecological model and empowerment theory (Zimmerman, 1995) | Educational, community-based participatory research | baseline, end of intervention | Subjective | Did not increase | Parent empowerment was measured | Did not increase |
| Geller 2012 (96) | n/a | QE | Not reported | USA | Older adult community housing sites | 21 | 72.24 (11.84) | 76 | Japanese | 23 | Definition not stated. The decisional balance sheet program provides basic health knowledge and empowers individuals to take personal control over their health. | Transtheoretical model | Educational | baseline, end of intervention | Subjective | Unable to determine | Not measured | n/a |
| Hoying & Melnyk 2016 (97) | COPE (Creating Opportunities for Personal Empowerment) Healthy Lifestyles TEEN (Thinking, Emotions, Exercise, Nutrition) Program | QE | Private | USA | Urban Middle School | 31 | 11.54 (0.62) | 65 | African American | 58 | Definition not stated. Cognitive behavior therapy (CBT) principles were utilized throughout the intervention. CBT philosophically aligns with Empowerment Theory, emphasizing agency, personal growth, and transformative change. However, CBT is more individually oriented and therapeutically focused, while empowerment theory often extends to collective and systemic levels of transformation. | Social cognitive theory | Educational with physical activity | baseline, end of intervention | Objective | Increased | Not measured | n/a |
| Huberty 2008 (98) | Women Bound to be Active | QE | Not reported | USA | College campus | 45 | 46.89 (11.33) | 100 | Non-Hispanic White | 91.1 | Women were encouraged to value themselves and make time for physical activity a priority. They were also encouraged to focus on what their bodies could do to improve their quality of life rather than their weight or clothing size. | Transtheoretical model, social cognitive theory | Educational | baseline, end of intervention | Objective | Increased | Not measured | n/a |
| Hyde 2020 (71) | Health Empowers You | QE | Private | USA | Elementary schools | 3294 | Not explicitly reported (4th grade) | 48.3 | Not reported | Not reported | Definition not stated. Empowerment is not defined. | Comprehensive school physical activity program | Policy change | baseline, end of intervention | Objective | Increased | Not measured | n/a |
| Keogh 2014 (72) | n/a | QE | Private | Australia | Residential Aged-Care | 34 | 83 (8) | 88.2 | Not reported | Not reported | Definition not stated. Empowerment was not defined; it was only mentioned once in the abstract. | None mentioned | Gaming | baseline, end of intervention | Subjective | Increased | Not measured | n/a |
| Kim & Cho 2013 (99) | EMPOWER Step Program | QE | Not reported | Korea | College campus | 37 | 19.18 (6.25) | 100 | Not reported | Not reported | The premise of this study is to empower exercise motivation, which is defined as "exercise development of the power latent within one and making a habit to live a successful life." E-Education, M - Measuring, P - Planning working, O - Outcome expectation, W - Workout keeping, E - Enjoying workout, R - Rewarding self | Social cognitive theory, self-determination theory, and others | Educational | baseline, end of intervention | Subjective | Increased | Not measured | n/a |
| Lin 2019 (82) | n/a | QE | Publicly funded | Taiwan | Not reported | 172 | 68.06 (8.44) | 71.6 | Not reported | Not reported | Definition not stated. Health empowerment is operationalized and defined as building confidence and enabling individuals to engage in self-management, solve health-related problems, and make informed decisions, thus promoting effective management of health conditions and achieving satisfactory health outcomes, particularly in populations with low health literacy. | Community-based participatory design strategies, health literacy framework | Educational, community-based participatory research | baseline, end of intervention, 6-month follow up | Subjective | Increased | Health empowerment via Health Empowerment Questionnaire | Increased immediately after the intervention but not the follow-up |
| Lindqvist 2014 (100) | n/a | QE | Not reported | Sweden | School | 53 | Not explicitly reported (adolescents) | 60.4 | Not reported | Not reported | Definition not stated. Instead, seminal empowerment was operationalized as a multidimensional construct that included individual influence over one’s life and participation in group and/or social activities. Five components of empowerment were emphasized. They included control, competence, confidence, contribution, and participation. | Social cognitive theory | Community-based participatory research, technology | baseline, end of intervention | Subjective | Increased | Not measured | n/a |
| Long 2020 (73) | n/a | QE | Private | USA | Primary Schools | 5882 | 8.69 | Not reported | African American | Not reported | Definition not stated. The term empowerment is used once in the abstract. The intervention fostered autonomy by allowing school-level leaders to select intervention components in alignment with the school's perceived needs. | None mentioned | Policy change | baseline, end of intervention | Objective | Unable to determine | Not measured | n/a |
| Manavi 2016 (101) | n/a | QE | Private | Iran | Not reported | 70 | 69.2 (3.4) | 81.4 | Not reported | Not reported | Definition not stated. The program aimed to empower participants to improve their physical mobility and ability to conduct their exercises independently. However, the intervention's approach to empowering participants is not clear. | None mentioned | Educational with physical activity | baseline, end of intervention | Subjective | Increased | Not measured | n/a |
| Melnyk 2013 (63) | COPE (Creating Opportunities for Personal Empowerment) Healthy Lifestyles TEEN (Thinking, Emotions, Exercise, Nutrition) Program | RCT | Publicly funded | USA | Secondary schools | 779 | 14.74 (0.73) | Not reported | Hispanic/Latino(a) | Not reported | Definition not stated. Cognitive behavior therapy (CBT) principles were utilized throughout the intervention. CBT philosophically aligns with Empowerment Theory, emphasizing agency, personal growth, and transformative change. However, CBT is more individually oriented and therapeutically focused, while empowerment theory often extends to collective and systemic levels of transformation. | Social cognitive theory | Educational | baseline, end of intervention | Objective | Increased | Not measured | n/a |
| Millstein 2016 (102) | Youth Engagement and Action for Health (YEAH!) | QE | Private | USA | After school programs, community groups, religious groups | 136 | 15.3 (2.73) | 73.1 | Hispanic/Latino(a) | 35.6 | Definition not stated. The intervention's conceptual model for youth advocacy for obesity prevention included individual-level factors (i.e., self-efficacy, self-esteem, empowerment, proxy efficacy, perceptions, behavior change skills, and barriers) influencing nutrition, physical activity, and advocacy skills. Social-level factors in the model included social support, social capital, and group norms, with peers, family, and neighborhood influences. Youth advocacy group characteristics included group structure and climate, group cohesion, collective efficacy, group resiliency, sense of purpose, levels of opportunities for responsibility, outcome efficacy, and decision-making processes. | Social cognitive theory | Educational | baseline, end of intervention | Subjective | Increased | Not measured | n/a |
| Morgan 2019 (61) | DADEE | RCT | Publicly funded | Australia | After hours at a primary school | 268 | Daughters = 7.7 (1.8) Fathers 41 (4.6) | Not reported | Not reported | Not reported | Definition not stated. The program aimed to combat societal gender bias that restricts girls' participation in sports and physical activity. Monitoring and control were integrated into the intervention. | Self-determination theory, social cognitive theory | Educational with physical activity, technology | baseline, end of intervention, 9-month follow-up | Objective | Increased | Not measured | n/a |
| Morgan 2022 (60) | DADEE | RCT | Publicly funded | Australia | After hours at a primary school | 341 | Daughters = 8.3 (1.8) Fathers = 42.0 (5.3) | Not reported | Not reported | Not reported | Definition not stated. The program aimed to combat societal gender bias that restricts girls' participation in sports and physical activity. Monitoring and control were integrated into the intervention. | Self-determination theory, social cognitive theory | Educational with physical activity, technology | baseline, end of intervention | Objective | Increased | Not measured | n/a |
| Neumark 2010 (103) | New Moves | RCT | Publicly funded | USA | Secondary schools | 356 | 15.8 (1.17) | 100 | African American | 28.4% (parents) | Definition not stated. The term self-empowerment is used, but it is not defined. The underlying program philosophy is that girls who feel good about themselves want to care for their bodies. | Social cognitive theory, transtheoretical model | Educational, coaching | baseline, 9-month follow-up | Subjective | Did not increase | Exercise Self-Efficacy | Increased |
| Noori 2021 (104) | n/a | RCT | Not provided | Iran | Workplace | 80 | 40.27 (13.07) | 100 | Iranian | 100 | Definition not stated. The main objective of this model is to empower people to cultivate an understanding of health promotion, and it includes four components: perceiving health threats, solving problems, educational participation, and evaluation | Based on the family-centered model of empowerment (Alhani, 2004) | Educational | baseline, 2-month follow-up | Subjective | Increased | Not measured | n/a |
| Onyegbule 2020 (66) | n/a | QE | Not reported | USA | Not reported | 28 | 35.75 | 100 | African American | 100 | Individuals are empowered to acquire new skills and employ resources to gain control. The empowerment model also unites marginalized communities to unite and utilize their strengths and resources to improve their quality of life. | Empowerment model (Moran et al., 2017), health belief model | Educational with physical activity | baseline, 12-month follow-up | Subjective | Unable to determine | Not measured | n/a |
| Pardo 2014 (105) | Sigue la Huella | QE | Not provided | Spain | Secondary schools | 682 | Children: 12-15 | 45.7 | Not reported | Not reported | Adolescents should take responsibility for their physical activity practice. | Social-ecological model, self-determination theory | Educational | baseline, 12+-month follow-up | Objective | Increased | Not measured | n/a |
| Pourfarzi 2023 (70) | n/a | RCT | Publicly funded | Iran | Web-based | 303 | 40-50 | 100 | Not reported | Not reported | Definition not stated. Empowerment is used two times in the manuscript, but it is not defined. Similarly, a theoretical framework is not used. | None mentioned | Educational | baseline, 3-month follow-up | Subjective | Increased | Not measured | n/a |
| Simangunsong 2017 (106) | n/a | QE | Not reported | Indonesia | Not reported | 70 | 40-45 | 100 | Not reported | Not reported | Definition not stated. According to the authors, empowerment means believing that premenopausal women have some control over their future health. | Premenopausal empowerment model | Educational with physical activity | baseline, end of intervention | Subjective | Increased | Not measured | n/a |
| Thøgersen-Ntoumani 2022 (112) | Can |  | Not funded | Austrailia | n/a | 153 | 28.31 (11.7) | 100 | Australian | 63.6 | Definition not stated. Empowerment was fostered through the autonomous motivation component of self-determination theory. | Self-determination theory | Media campaign | baseline, end of intervention | Subjective | Did not increase | Not measured | n/a |
| Tucker 2017 (65) | Health-SmartChurch (HSC)Program | QE | Private | USA | Church | 70 | 35-49 | 81.4 | African American | 100 | Definition not stated. Health self-empowerment theory recognizes that health-smart behaviors are impacted by social, environmental, and economic conditions that are often intractable. Therefore, health behavior change is influenced by five key modifiable, cognitive behavioral, and self-empowerment-oriented variables that enable these behaviors to occur under whatever conditions (e.g., economic conditions). These key variables are (a) healthy self-efficacy, (b) healthy motivation, (c) healthy self-praise, (d) healthy responsibility/ knowledge, and (e) active coping strategies for managing anger, stress, and depression. Furthermore, this theory asserts that positive health behavior changes result when individuals are empowered to identify barriers to health behavior change and address these by increasing awareness and utilization of the variables. | Health self-empowerment | Community-based participatory research, technology | baseline, end of intervention | Subjective | Increased | Not measured | n/a |
| Tucker 2019 (64) | Health-Smart AME | QE | Private | USA | Church | 321 | 54 (16) | 0.648 | African American | Not reported | Definition not stated. Health self-empowerment theory (see Tucker 2017) was operationalized. | Health self-empowerment | Community-based participatory research, technology | baseline, end of intervention | Subjective | Increased | Not measured | n/a |
| Wani 2024 (107) | Ath Waas | QE | Not funded | India | Not reported | 67 | 31-40 | 0.919 | Not reported | Not reported | Definition not stated. The approach was akin to patient empowerment. "The empowerment approach “redefines the professional’s role as a collaborator [where] participants have an active role in the change process.” | Social learning theory, peer support, and empowerment theory (no reference) | Community-based participatory research, technology | baseline, end of intervention | Subjective | Increased | Not measured | n/a |
